# Academic (Under)achievement of Intellectually Gifted Students in the Transition Between Primary and Secondary Education: An Individual Learner Perspective

**DOI:** 10.3389/fpsyg.2019.02533

**Published:** 2019-11-13

**Authors:** Katelijne Barbier, Vincent Donche, Karine Verschueren

**Affiliations:** ^1^Department of Training and Education Sciences, Faculty of Social Sciences, University of Antwerp, Antwerp, Belgium; ^2^Faculty of Psychology and Educational Sciences, School Psychology and Development in Context, KU Leuven, Leuven, Belgium

**Keywords:** Achievement Orientation Model, gifted students, underachievement, self-regulation secondary education, qualitative research and analysis, self-regulation

## Abstract

In the last decade, the Achievement Orientation Model (AOM) of Siegle and McCoach has often been used to quantitatively explore different pathways for academic achievement among intellectually gifted students in educational settings, mostly in secondary education. To study the dynamics of the different components in the AOM, we further examined the inhibiting and facilitating factors associated with academic achievement as experienced by well-performing and underperforming gifted students. Because the transition from elementary to secondary education is a crucial phase for intellectually gifted students, we selected students from the 7th and 8th grade, using purposive sampling. Six gifted students, three well-performing and three underperforming, from two different high schools participated in in-depth interviews. By capturing the lived experiences of six intellectually gifted students in this study, we were able to get more insight into the complex processes that relate to students’ (dis)engagement and (under)achievement in school. The findings underline the value of the AOM and stress the importance of taking learner perceptions into account.

## Introduction

Since the 19th century, attention has been paid to cognitive talent in the scientific literature (Gagne, 1985; Cravens, 1992). With the rise of research on giftedness, the problem of underperforming gifted students in education was also raised (Dowdall and Colangelo, 1982). Lack of motivation is seen as a possible explanation for underachievement among cognitively gifted students (Whitmore, 1986; Rea, 2000; Cakir, 2014; White et al., 2018). Siegle and McCoach (2005) developed the Achievement Orientation Model (AOM). This model is grounded in previous research on motivation and intellectual giftedness and shows the different factors that determine whether cognitively gifted students (under)achieve. The AOM points at a number of factors related to motivation, task engagement and achievement, namely self-efficacy, goal valuation, environmental perceptions, and self-regulation. Although research on the AOM has revealed the importance of interactions with teachers (Siegle et al., 2014), and parental involvement (Rubenstein et al., 2012; Brigandi et al., 2018) and the benefits of homogeneous grouping with like-minded peers (Brigandi et al., 2018), some gaps can be pointed out in the existing literature concerning the AOM.

Most research regarding the AOM has used a quantitative approach, and as a consequence little is known about how students perceive the interplay of these factors, or how these factors interact and depend on each other in specific educational contexts. Qualitative research enables delving deeper into students’ perceptions of this complex process. There is one retrospective qualitative study on the AOM (Siegle et al., 2014), in which former students looked back on their high school experiences. However, retrospective studies have their pitfalls, as students may not accurately recall past events (Beckett et al., 2001). The study of Brigandi et al. (2018) also uses qualitative research, but this study is limited to one aspect of the AOM, namely environmental perceptions. Gaining more insight into the lived experiences of the students is important to grasp the complexity of the process of motivational development. A limited number of quantitative studies have already pointed out the relevance of studying the AOM during the early school career of cognitively gifted students (Rubenstein et al., 2012; Ritchotte et al., 2014). In addition, other studies have accentuated the transition from elementary to secondary education as a crucial phase for motivational development, particularly for intellectually gifted students (Snyder and Linnenbrink-Garcia, 2013; Coelho and Romao, 2016; Evans et al., 2018). However, qualitative research with a focus on the first grades of secondary education is missing in research concerning the AOM. Furthermore, the AOM has been mainly studied in American school contexts and it remains unclear if the AOM can also be applied to other educational contexts. It would be interesting to also have more context and time specific empirical research on the AOM in a different educational context.

Gaining insight in perceptions of cognitively gifted students in the transition from elementary to secondary education is important for further theory development. We want to take the lived experiences of the subjects on the one hand and the intra individual mechanism they describe on the other hand into account. Meaning that we look at how the process of (under)achievement (conceptualized by the AOM) is actually put together by the students (Byrne and Ragin, 2009). By getting grip on these aspects we are able to gain a deeper understanding of the AOM. This deeper understanding of the students experiences is important to further develop the theory of the AOM. The general aim of this study is to use the AOM as a theoretical lens, to look into in the interplay of factors situated in the model, and to unravel the complexity of the process that leads to (under)achievement and school (dis)engagement of cognitively gifted students after transitioning to secondary education. By using a qualitative research perspective, we aim to increase understanding of the perceived realities of gifted students (Holloway, 1997; Savin-Baden and Howell, 2013), and also to reveal facilitating or hampering factors (from the AOM) for intellectually gifted students’ engagement and achievement as described by the respondents themselves.

In what follows, we outline our interpretation of giftedness, situate the AOM within the literature and give further explanation on the need for more qualitative research on motivational development in gifted students and the role of self-efficacy, goal valuation, environmental perceptions, and self-regulation.

## Gifted Students

Giftedness is a term commonly used in research. However, there is no widely accepted definition of the concept, and assumptions about and criteria for giftedness differ between theoretical models (Gagne, 1985; Sternberg and Zhang, 1995; Renzulli, 1999; Heller et al., 2000; Sternberg, 2003; Siegle and McCoach, 2005; Subotnik et al., 2011). Despite these differences, common features can be found in the models: there are multiple domains of giftedness (e.g., artistic, athletic, cognitive). Also, there is a distinction between outstanding abilities, on the one hand, and fully developed forms of outstanding mastery, on the other hand. Most models are *developmental* in nature, meaning that they assume that outstanding cognitive abilities are gradually transformed into (outstanding) academic performance. In addition, *environmental and personal* factors play an important role in either facilitating or hampering the transformation or development of abilities into academic performance. Depending on the specific model, these factors are conceptualized differently. Across models, then, gifted students are students who excel in a certain domain, taking into account the environmental and personal factors. We therefore address gifted students as ‘intellectually gifted’ or ‘cognitively gifted’ in this study.

When talking about ‘intellectually gifted students,’ underachievement is a frequently mentioned phenomenon within educational contexts (Rubenstein et al., 2012; Snyder and Linnenbrink-Garcia, 2013). Ritchotte et al. (2014) describe underachieving intellectually gifted students as ‘a loss of potential for society’. The most general definition of underachievement or underperformance refers to the discrepancy between ability and performance. Gifted underachievement is, just like the concept of giftedness, very difficult to define. Dowdall and Colangelo (1982) found fifteen different definitions of ‘underperforming gifted individuals.’ The lack of agreement on the concept also contributes to the lack of insight into the problem of underachievement (Schultz, 2002). Reis and McCoach (2000) formulated a definition of underachievement that integrates different aspects of the range of definitions and which is used in this study:

Underachievers are students who exhibit a severe discrepancy between expected achievement (as measured by standardized achievement test scores or cognitive or intellectual ability assessments) and actual achievement (as measured by class grades and teacher evaluations). To be classified as an underachiever, the discrepancy between expected and actual achievement must not be the direct result of a diagnosed learning disability and must persist over an extended period of time. Gifted underachievers are underachievers who exhibit superior scores on measures of expected achievement (i.e., standardized achievement test scores or cognitive or intellectual ability assessments). (p.157)

Within the group of underperforming intellectually gifted students, we can distinguish between absolute underachievers and relative underachievers (West and Pennell, 2003). In the first category, the student scores below the general level of the class. Within the second category, students do not perform below the class norm but does perform below his or her own abilities. The latter students often stay ‘under the radar,’ because they still perform relatively ‘normally’ compared with the rest of their classroom peers (Subotnik et al., 2011). Scholars argue that intellectually gifted students are likely to underachieve when they lack motivation (Rea, 2000; Morisano and Shore, 2010; Cakir, 2014). In the review of White et al. (2018) nine articles on intellectually gifted underachievers were analyzed, and motivation was frequently reported as being lower among cognitively gifted underachievers when compared to cognitively gifted achievers. This difference has been found across a broad variety of self-reported indicators of motivation (e.g., learning goal orientation, achievement ambition and joy for learning). We can therefore conclude that intellectual gifted underachievement is often linked to different types of motivational problems.

## Achievement Orientation Model

It is clear from previous research that the underachievement of cognitively gifted students is closely related to motivational deficits. Previous research on motivation of under- or well-performing intellectually gifted students suggests that enhancing the engagement and achievement of these students can be a complex process, as many other factors come into play. If we aim to shed further light on the role of inhibiting or facilitating factors that influence the motivational development of intellectually gifted students, the Achievement Orientation Model (AOM) provides a useful theoretical lens.

Previous research on motivation and (under)performing cognitively gifted individuals formed the basis for the AOM (Siegle and McCoach, 2005). Self-efficacy theory (Bandura, 1986), expectancy-value theory (Eccles and Wigfield, 1995) and person-environment fit theory (Lewin, 1963) are underlying theories incorporated into the AOM. In what follows, we will discuss the most important components of the AOM.

**FIGURE 1:**
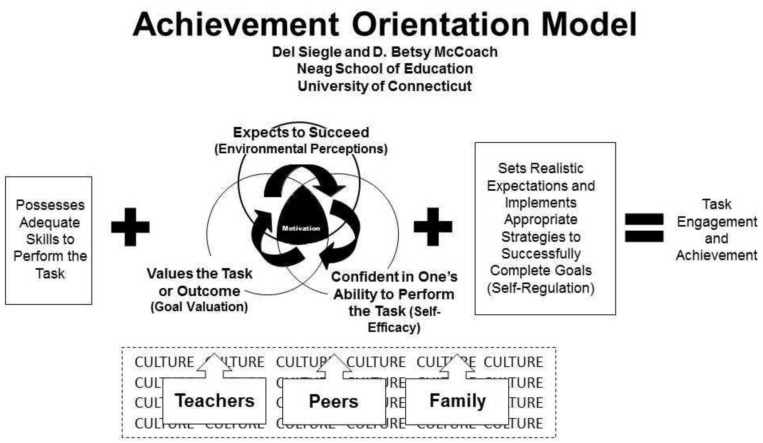
Achievement Orientation Model. Reprinted with permission of Del Siegle.

The AOM (see Figure 1) distinguishes three domains that are important components of the motivation of intellectual gifted students: self-efficacy, goal valuation, and environmental perceptions of support (Siegle and McCoach, 2005). Motivation is a result of the interplay of these three factors, which then enables the students to self-regulate their learning and engage and achieve in school tasks. Through this model we can gain insight into the process and influencing factors that lead to better performance. At the same time we can expect that if these components are not present, the student’s motivational development will not be optimal, which can lead to underachievement.

First, the domain of *self-efficacy* entails the students’ beliefs that they have the necessary cognitive skills to be successful. Siegle and McCoach (2005) stated that when students have high competence beliefs, they feel efficacious. When intellectually gifted students have doubts about their competence, this can activate maladaptive coping mechanisms (Snyder and Linnenbrink-Garcia, 2013), implying that they use underperformance as a means to avoid situations in which they might fail.

The second domain is *goal valuation*. This refers to the extent to which students consider certain tasks as worthwhile. The aspect of goal valuation is divided into three factors: the intrinsic value (a student’s interest in a task), the utility value (the meaningfulness of a task) and the attainment value (the importance students attach to the task as it relates to their conception of their identity and ideals). According to the AOM, students can be motivated by one or more of these factors (Siegle and McCoach, 2005).

The third domain deals with the students’ perceptions of support in their learning environment (*environmental perceptions*). The degree of support that intellectually gifted students experience within their environment influences their academic attitudes and achievements. This support or lack thereof can be experienced, for example, through expectations from parents and teachers, the interaction between students, teachers, and parents, and through events at home or at school. It is possible that students who do not experience their environment as supportive have problems with the authority of teachers or school staff (McCoach and Siegle, 2003).

Next to the three domains, *self-regulation* is also an important component in realizing achievement. Self-regulation contains three elements: self-management, personal standards, and self-monitoring (Siegle and McCoach, 2005). Self-management refers to the strategies and skills required to process large amounts of subject matter. These competencies include, among others, time management and study skills. Personal standards entails what the students think warrants ‘good enough’ performance, and includes setting realistic expectations. Self-monitoring includes, among other things, monitoring of distraction and being able to delay satisfaction (e.g., first carrying out a less enjoyable task and only then completing a satisfying fun task). When the students are motivated and has the skills to self-regulate himself, he can engage and achieve in school tasks.

In addition to self-efficacy, goal-valuation, environmental perceptions and the interaction with self-regulation, teachers, curriculum at school, attitudes of and activities with friends, and the home situation are all assumed to influence this process (Siegle and McCoach, 2005).

It is clear from the AOM that many different factors are assumed to play an influential role in the engagement and achievement of intellectually gifted students. Viewed from this model, when students have a positive attitude toward the three areas: self-efficacy, goal valuation and environmental perceptions of support, and they also have adequate self-regulation skills, this is associated with more task engagement and higher achievement. A less optimal path is likely when students face problems relating to the different domains (self-efficacy, goal valuation, environmental perceptions and self-regulation). According to this model, there is no predefined path for (under)achievement; in many cases it will be a combination of positive and negative influences that have a cumulative impact on engagement and achievement (Siegle and McCoach, 2005).

## This Study

The transition from elementary to secondary education is a crucial phase in the school career, particularly for intellectually gifted students (Snyder and Linnenbrink-Garcia, 2013; Vaz et al., 2014; Coelho and Romao, 2016; Evans et al., 2018). When primary education does not offer sufficient challenge or opportunities to develop study skills, this can be problematic in secondary education. For example, we can find a low preference for self-regulated learning (SRL) strategies among intellectually gifted students. These students tend to do well in school for a long time without using learning strategies or self-regulating their learning (Stoeger et al., 2014, 2015) and thus fail to recognize the usefulness of such strategies. When the course content suddenly becomes more challenging in the transition to secondary school, and students cannot rely on SRL skills, they are likely to fail in achieving good grades. Also, if students were not challenged academically in the past, they can adopt an attitude that assumes they will have to make little effort to achieve a satisfactory result (Snyder and Linnenbrink-Garcia, 2013). Because the transition to secondary school is critical for the reasons enumerated above, this study will be conducted in Grade 7 and 8.

In the past, several studies have already been carried out that have demonstrated the usefulness of the AOM for explaining intellectually gifted students’ motivational development and the outcomes of this development (Rubenstein et al., 2012; Ritchotte et al., 2014; Siegle et al., 2017; Brigandi et al., 2018). These studies have mainly been quantitative in nature. Because the lived experiences of the students are absent in previous research on the AOM, a qualitative approach was chosen for the current study. As stated in the introduction, there are two qualitative studies on the AOM (Siegle et al., 2014; Brigandi et al., 2018), both which are retrospective or focused on the upper secondary grades. This study adds to this previous research, as students were interviewed about their secondary school experiences while they were in the first years of secondary school, a key transition period that has not been studied so far. By opting for qualitative research and focusing on this particular transition, we can get a richer picture of the experiences of the students and extend previous insights (Holloway, 1997; Savin-Baden and Howell, 2013). There is no assumption of an objective, unambiguous truth in the process of (de)motivation and (under)achievement, but it is assumed that there are multiple realities, formed by individual perceptions (Savin-Baden and Howell, 2013).

In this qualitative study we will focus on the core components of the AOM. No data were gathered on the students’ ‘home,’ ‘peers’ and ‘school’ (e.g., the curriculum at school, the professionalization level of the teachers, the attitudes of friends or the social economic status of the family). Accordingly, we will only look at the interplay between the four main components of the AOM (i.e., self-efficacy, goal valuation, environmental perceptions and self-regulation) and how this interplay shapes students’ task engagement and achievement in education, as experienced by the students. We opted for purposeful sampling and using a case based approach, because this enables us to provide in-depth descriptions of differently performing intellectually gifted students (Miles et al., 2014; Corbin and Strauss, 2017). The case-based approach will help us understand the relevance of AOM in specific educational contexts and time (transition primary to secondary).

In this study, we have two research aims:

(1) Identify the components central to the AOM (i.e., self-belief, goal valuation, environmental perception, and self-regulation) in the students’ lived experiences.
(2) Explore the factors that can hamper or facilitate student engagement and achievement of intellectually gifted students, and their complex interplay, as experienced by the students.

## Methodology

### Sample

To attain informational richness, a purposive sampling strategy was selected, resulting in the selection of 6 intellectually gifted male students from the first and second year of secondary education. Seven secondary schools in the Dutch speaking part of Belgium (Flanders) were contacted, of which only four schools were willing to cooperate. One of these four schools indicated that they did not have students who fit the profile for the study. At another school, the parents did not give permission for their child’s participation in the study. The remaining two schools were willing to cooperate: School X and school Y. In School X, there was no specific attention for intellectually gifted students. The school leaders indicated that they were aware of this shortage and therefore they were enthusiastic about participating in this study. At school Y, there was an enrichment pullout program, in which intellectually gifted students learned how to work systematically while stimulating metacognition, motivation and well-being. Intellectually gifted students in school Y were not obliged to participate in this enrichment project.

After informing the schools about our desired respondents (i.e., defining intellectually gifted students), students were selected by the school counselors, using a multi-informed approach. The school counselors and care teams had various conversations with the students, their parents and their teachers. Also they made an analysis of the students’ academic performance in elementary education. Based on the academic performances, the various conversations, and taking into account the indicators of students’ intellectual giftedness, (e.g., a high capacity for reasoning and problem solving or an excellent memory) they nominated the students. Furthermore, the school counselors were asked to fill out a questionnaire, to substantiate the identification of the children as intellectually gifted (e.g., When it was established that the student was gifted? How was this determined? Do you think this student is currently performing according to his/her capabilities or is he or she underperforming? Why?). Based on the conversations with the teachers and inspection of current academic performances, the school counselor stated that the underperforming students were not absolute but relative underperforming. Of the six selected students, three students were well-performing students and three were underperforming students in school. Only one student had a formal diagnosis of intellectual giftedness (based on an intelligence test). One student had been accelerated by one school year in primary school; another had followed a few courses at a higher grade level in primary education. Table 1 shows the most important characteristics of the respondents in a more structured way. In order to guarantee the anonymity of the respondents, other names are used.

**TABLE 1:** Background characteristics of the different respondents.

**Respondent**	**Vince**	**Sebastian**	**Thomas**	**Nick**	**Jack**	**Liam**
Age	11	12	13	12	12	12
Grade (sec. ed.)	7	7	8	7	7	7
Gifted based on…	Beliefs	Beliefs	IQ-test	Beliefs	Beliefs	Beliefs
School performance	UP	UP	UP	GP	GP	GP

*UP refers to a relative underperformance at school. GP refers to a good performance at school.*

All of the selected students were attending the first or second year of secondary school in Flanders and were following an academically oriented study track^1^ (i.e., classical studies like Latin). Most of the participants were 12 years old. We have no information on their socioeconomic status. Both well-performing and underperforming students were included in this study. After informing the parents, ensuring them of confidentiality and anonymity and obtaining a written consent from both parents and students, six students agreed to be interviewed.

### Instrument

To elicit students’ personal experiences, a semi-structured interview guide was used with open-ended questions based on the key concepts of the AOM (see Supplementary Appendix). The questions were designed in such a way that the different themes of the AOM were discussed. The categories in the interview guide were environmental perceptions, goal valuation, self-beliefs (including self-efficacy) and self-regulation. Some questions were formulated in an open way, e.g., “Do you find it important to perform well at school? Why?” Expected themes that could be addressed with this question are task meaningfulness, self-regulation (personal standards) and environmental perceptions. Others were more focused on one theme: e.g., “When do you think a task is useful?” (goal valuation: utility value). In addition, a specific question was asked about self-monitoring (self-regulation) during the interview. Namely: “Which statement is most relevant to you? And why? (1) I prefer to first do tasks that I like. The tasks that I like less, I postpone as long as possible. (2) I always complete the less fun tasks first; afterward I can complete the tasks that I like to do.” The aim of using semi-structured interviews was to give the respondents the opportunity to express their opinions and ideas in their own words so that they could determine the structure of the interview to a large extent (Savin-Baden and Howell, 2013).

### Procedure

A pilot interview was first conducted with a non-gifted 12-year-old student. The aim of the pilot study was to determine whether the guideline would work for the age group (is all terminology understandable? Does the pace of the interview allow open conversation, is the interview not too long or do we notice other issues of incomprehensiveness of the questioning?). Afterward, the structure of the guideline was examined and optimized. The interviews were conducted in the spring of 2016 and lasted for 30–45 min. All interviews took place in a quiet room at the students’ school. The researcher recorded each interview digitally. The first author conducted the interviews and the analyses. In addition, peer debriefing was used, involving regular discussions between the first author and the other two authors regarding the process, the choices that were made and the conclusions. Peer debriefing contributes to the validity and reliability of the research (Savin-Baden and Howell, 2013).

This study was carried out in accordance with the Social and Societal Ethics Committee of the University of Leuven. The authors declare that the research was conducted in the absence of any commercial or financial relationships that could be construed as a potential conflict of interest. The raw data supporting the conclusions of this manuscript will be made available by the authors, without undue reservation, to any qualified researcher.

### Analysis

First, the interviews were transcribed verbatim. Afterward, the quality was checked by reading the text and listening again to the audio fragments. All misunderstandings were corrected during the second round. The interviews were transcribed in Dutch, only in the last phase of writing this article, the quotes were translated into English. To minimize the loss of meaning inherent in the translation process (Hammersley, 2010), a bilingual researcher made the translation.

To analyze the interview data, thematic analysis was used, using the program Nvivo11. We opted for a mixed coding approach, meaning that both deductive and inductive coding were used (Marshall and Rossman, 1995; Bruce, 2000). In a first phase, deductive coding was used, based on a coding scheme based on the AOM (see Table 2 for the complete coding scheme). In addition, each code received a positive or negative value (value coding). In a second phase, we added new codes during inductive coding to make sure all the topics addressed by students were coded. First, a within-case analysis was performed: in which each case was examined separately. By using a case-based research approach (in contrast to a variable centered approach), we try to conceptualize the person as an integrated totality, rather than as a summation of variables (Byrne and Ragin, 2009). Afterward, we made a between-case analysis: which themes and obstructing/facilitating factors are discussed across the different interviews regarding the AOM? In order to accomplish the second aim: ‘To explore the factors that can hamper or facilitate engagement and achievement of intellectual gifted students, as described by the students.’, we compared the codes of well- and underachieving students. To increase the reliability of the coding, we asked a research assistant to code a sample of our data, based on a given coding scheme with the four broad categories and the sub codes. Cohen’s κ indicated a fair to good agreement: κ = 0.65 (Fleiss, 1971). In addition, this step was also discussed via peer debriefing with the second and third author.

**TABLE 2:** Coding scheme and number of fragments (N).

**Code**	***N***	**Value coding**		
**Goal valuation**	110	The respondent indicates that he considers learning contents as meaningful and/or interesting.	OR	The respondent indicates that he does not considers a certain task as meaningful and/or interesting.
***Subcodes:***				
*- intrinsic value:*				
*- challenge*				
*- utility value*				
*- attainment value*				
**Self-efficacy**	28	The respondent indicates that he believes he has the necessary cognitive skills to be successful	OR	The respondent indicates that he does not believes he has the necessary cognitive skills to be successful or he indicates that he considers his peers, his family or his teachers as non-supportive.
***Subcodes:***				
*- fixed mindset*				
*- labeling*				
**Environmental perceptions**	89	The respondent indicates that he thinks that his environment (teachers, peers, parents,…) believes in his capacities or he indicates that he considers his environment as supportive.	OR	The respondent indicates that he thinks that his environment (teachers, peers, parents,…) do not believes in his capacities or he indicates that he considers his environment as non-supportive.
***Subcodes:***				
*- home (parents)*				
*- teachers*				
*- peers*				
**Self-regulation**	82	The respondent indicates that he can set realistic expectations and/or can implement appropriate strategies to successfully complete goals.	OR	The respondent indicates that he cannot set realistic expectations and/or can’t implement appropriate strategies to successfully complete goals.
***Subcodes:***				
*- self-management*				
*- personal standards*				
*- self-monitoring*				
*- transition*				
*- elementary-secondary education*				

## Results

First, we use the AOM as a theoretical lens to gain insight into factors inhibiting and facilitating engagement and achievement among intellectually gifted students. Next, we present which factors seem to be likely influential for the engagement and achievement for all intellectually gifted students and we zoom in on the difference between well- and underachieving students. As an example, we illustrate how the interplay between the students’ engagement and achievement and these factors was present in the data and perceived by a good achieving versus an underachieving student.

### The Achievement Orientation Model

Concerning the first research aim, a first observation was that all components of the AOM were described by the respondents. One component was discussed in a more superficial way, namely self-belief (including self-efficacy), meaning that students answered it in one or two sentences only. The other components were discussed in detail (goal valuation, environmental perceptions and self-regulation), meaning that students talked more extensively about their experiences of these components. When looking at the frequency of the different codes (Table 2), this confirms the fact that self-belief was discussed less by the students.

The usefulness of a task, the intrinsic motivation to perform a task or the will to perform well (*goal valuation*) was often discussed related to motivation, task engagement and achievement:

Sometimes with those definitions I also think: “What good is it that you know those definitions literally?”. It is just good if you understand the definitions and can do the exercises, instead of studying those definitions by heart. Later with your job or with an application they will never ask what the exact definition of a right angle is. I don’t think that is useful to know, but I will learn them anyway. (Liam, GP)

In addition, the respondents indicated if they believed in their own capacities or not, but in a more superficial way (*self-efficacy*): *“I am confident. I experience being smart as something positive.” (Jack, GP) or “It is not that I say,” “Yes, I have talent!”. No, it is not that I say, “I am super smart.” (Sebastian, UP).* Students also talked about the extent to which they thought their environment believed in them and supported them (*environmental perceptions*) and how that influences their motivation, task engagement and achievement.

Yes, that’s because I had friends then who played computer games all the time. And then I studied much less… and then they said: “No, we are not your friends anymore. Just go away. (…) I am still bothered by being bullied. There are a few who say that I study a lot and they laugh at me. Because my mom makes me study a lot. (Vince, UP)

The respondents talked profoundly about their self-regulating skills (including self-management, personal standards, and self-monitoring) and how this influences their motivation, task engagement and performance:

I learn my Latin vocabulary. Suppose you have to learn 300 words for your exam by heart…then I try to plan it as good as possible so I don’t have to do everything at the last minute. That was a bit of a problem at the beginning of the year. I did everything at the last minute and I thought it would work out, but in the end it turned out that things didn’t work out so well. So now I try to plan everything as good as possible. (Liam, GP)

### Overall Inhibiting and Facilitating Factors

For the second research aim, it is clear that a multitude of factors (self-efficacy, environmental perceptions, goal valuation, and self-regulation) are related to task engagement and achievement. Although the perceived interplay of factors that lead to (under)achievement is different for each respondent, there are similarities that are present across the six respondents as well as within the distinct groups of well-performing versus underperforming students. Next, we detail the inhibiting and facilitating factors for academic (under)achievement according to the intellectually gifted students in our sample. Afterward we will discuss two cases to show in detail the interplay between the different components.

#### Self-Regulation

There was a clear difference in the monitoring of delayed satisfaction between well-performing intellectually gifted respondents and the under-performing gifted respondents. All high-performing cognitively students indicate that they would choose to first complete less enjoyable tasks, after which they would engage in the tasks they would like to do: *“It’s stupid to finish my homework with tasks that I don’t like much. Then you actually end your work with a negative feeling, because you didn’t like it. But suppose you finish with a nice task, then you will find that completing those tasks is not a waste of time”* (Liam, GP).

All underperforming respondents opted for the option where they could first complete the fun tasks, only thereafter focusing on the tasks they did not like: *“I’d rather do something fun than do something stupid. So I just try to postpone the stupid tasks.” (Sebastian, UP).* Because they want to complete the fun tasks immediately, we can say that these respondents have difficulties with delayed task gratification. Therefore, a lack of self-regulation might be a hampering factor, and being able to postpone a more appealing task appears to be a facilitating factor, for achievement and task-engagement.

I’ve always had a harder time learning. I just can’t do it. I sometimes don’t know how to write something down. In the lower classes it all went smoothly and also at the beginning of the school year, but now the subject matter is more difficult. I didn’t study much in the past and now I just have to learn how to study something, and how to study well. (Sebastian, UP)

Sebastian’s example confirms the AOM’s assumption that a lack of study skills can lead to underachievement when the subject matter becomes more challenging (Siegle and McCoach, 2005). Lacking study skills can therefore be an inhibiting factor to achievement. Interesting in this aspect of self-regulation is the role that the school plays. All six participants indicate that the school tries to teach them SRL strategies. However, when the students’ experiences were probed about this offer, all six of the respondents were not interested in the school’s program concerning self-regulation strategies: *“Yes ‘learning how to learn’ does exist in our school; this session is every Tuesday, one day a week. But that’s for slightly lesser smart, or average students.” (Nick, GP)*

#### Environmental Perceptions

The between-case analysis shows that a distinction is made between support from parents, teachers, and friends. High-performing respondents point out that they experience their environment, both at home (parents), at school, and with their friends mostly as supportive. This does not necessarily mean that school environments meet their needs. The majority of the respondents spoke about the hampering effect of the lack of challenge and the lack of interesting tasks at school.

I thought it was about Romans and stuff like that, and how it used to be in the past. But it’s about the different types of people. Yeah, I like that less. (…) If we have to follow the lessons with the whole class all the time, it goes so slooowly. (…) I don’t think I’m being challenged enough. (Sebastian, UP)

In addition, the respondents spoke multiple times about the transition from elementary to secondary education. This transition was not necessarily a positive experience for the respondents. They indicated that elementary education was more challenging or that secondary education was more challenging in terms of social aspects (different teachers, large school, etc.).

[In secondary school] You also have to deal with every teacher, which is also a challenge in some cases. In primary school you had one teacher. Sometimes you also have to do all the different tasks and remember what each teacher said, that is sometimes…yeah…(Liam, GP)

Among the underperforming respondents, most of them did experience at least one of their environments as non-supportive. Thomas for example, points out he sometimes clashes with certain teachers. It can be said that Thomas has a negative attitude toward the school and the teachers, which can possibly lead to underperformance.

The teacher gets really mad, then she hits on the table and shouts: “You’re not going to make me mad again, are you?” But I don’t remain silent. Not that I start to shout, but I do answer. She can’t stand that and then she gets even worse. (Thomas, UP)

#### Goal Valuation

Wanting to attain good grades is the main motive for all respondents to put forth effort in school, regardless of their performance, and this motive is clearly a facilitating factor for achievement at school. Although underperforming students state that this is a facilitating factor in their school achievement, they nonetheless fail to academically engage and achieve:

The grades. If I reach 83 (out of 100), I am not satisfied with that, but it will do the job. I learn for the grades I get. (…) I don’t always have motivation and I drag my feet. (Thomas, UP); I think school is important to get good grades. (Jack, GP)

In the data we see that the respondents have both positive and negative *intrinsic motivation* experiences, regardless of their performance. Thus, for these respondents, intrinsic value is not a key impeding or facilitating factor in their (under)achievement. Other factors are clearly at stake, as good performing students still achieve when they are not intrinsically motivated, and underperforming students underachieve despite being intrinsically motivated.

Most respondents expressed a lack of intrinsic motivation several times during the interviews. Some found the lessons boring or too slow, others experienced a lack of challenge. Also incorrect expectations of courses sometimes led to a lack of motivation among these students.

An easy task is pretty boring. In the technology-class, it often happens that we get a graded task that we need to complete. We get half an hour for the task and I am done after 10 min. (Vince, UP)

I am not that interested in religion and I find Dutch quite easy. (…) Math is my best subject, but this year I think it is all a bit slow. Yes…really. I prefer a bit more challenge. (…) (Nick, GP)

All respondents made statements that show that they are intrinsically motivated for some courses, as interesting subject matter was pointed out as a main reason for motivation. To a lesser extent, the role of the teacher was also discussed when talking about interesting courses*:*

In the past, that course actually was a course where I could really easily get high grades without doing much effort, but now I really participate and work in class. And listen to know more about history. I think it’s too bad that it is only 1 h a week. (Vince, UP)

Natural sciences, like I said, I really like this. I just find it interesting and the teacher also gives nice lessons by showing experiments and such. (Liam, GP)

#### Example: The Interplay of Facilitating and Hampering Factors

The within-case analyses reveals that every student shows a unique and complex interplay of facilitating and hampering factors. To illustrate the complexity and uniqueness of the processes that may lead to (dis)engagement and (under)achievement we provide a more in-depth description of two cases, Nick and Thomas, respectively a high achieving and underachieving intellectually gifted student.

Nick (GP) believes in his cognitive skills and knows that he can complete a task successfully (*self-efficacy*). He has high intrinsic motivation, finds most of the subject matter useful and has a drive to perform well (*goal valuation*): *“I just think it’s important that you learn, so I just do it.”* Nick points out that his parents don’t help him anymore with his homework. Still, he experiences his environment as positive: his parents, but also his friends and his school provide support.

Since this year my parents no longer help me review the study content. They said after the sixth grade: “Now you have to study independently. Later the study material will become so large that we can’t help you review.” (…) My parents fully support me in my school work. (Nick, GP)

Yet there is not a complete match between himself and the school (*environmental perceptions*). Although he points out the subject matter is difficult in secondary education, he preferred elementary education because he could work at his own pace. The lessons in secondary education are too slow for him and he prefers to work independently. He likes to research things himself and prefers tasks where he needs to think, something which is not always present in secondary education. Nick knows how to regulate his own learning and how to learn subject matter. His self-regulation skills are developed enough to succeed in difficult tasks.

[About a project for intellectually gifted students] It is great, but I really liked it more during elementary school. We really worked together there to discover things, and now it’s not really difficult. It’s just looking up things and you learn a little bit, but I don’t have to think it through. You learn, but you don’t think. (Nick, GP)

We see that almost all factors postulated in the AOM have a facilitating effect on Nick. Nick beliefs in himself (*self-efficacy*), has a lot of intrinsic motivation and finds most tasks meaningful (*goal valuation*) and is able to set realistic expectations and regulate his own learning (*self-regulation*). Only one aspect of a domain, namely *environmental perceptions*, has a minor impeding effect. The interplay of the facilitating effects ensures that Nick is engaged with learning.

Thomas (UP) knows he has a lot of talent, but prefers to keep this to himself. He also indicates that he is not making optimal use of his cognitive skills. He points out that if he worked harder, he could perform better (*self-efficacy*). Thomas has a lack of intrinsic motivation (*goal valuation*). He thinks school is useful, but he cannot make the effort to perform according to his abilities.

Yes, school is important. Especially for later, to have a diploma and find a job. Here you just learn about the basics of everything that you will do later. (…) On the one hand, it is indeed motivating, but it does not motivate me enough, and I still get good grades even though I am not doing anything for it.

Next, he experiences both his home environment and his school environment as non-supportive (*environmental perceptions*). Thomas’ father recently passed away. According to Thomas, this event has an impact on the motivational process and clearly contributes to his lack of motivation. He does have good friends whom he can count on. He also points out that he does not get the support he needs at school. Thomas refers to the fact that he thinks the teachers are not aware of his high abilities and therefore do not consider this or support him: *“It’s not that my grades at school are bad, so the teachers don’t worry. A lot of teachers don’t even know [that I’m gifted].” (Thomas, UP)*

Thomas has sufficient self-regulation skills to complete a task successfully. However, he sometimes chooses not to use these skills if he doesn’t feel like it: *“If we have a test of a language course and I don’t like it, then I’ll just read instead of write.”*

We see that various elements have an impeding effect on Thomas. He beliefs that he can do better (*self-efficacy*) and he states that having the necessary *self-regulation* skills, but he experiences both his home and school environment as non-supportive to his learning (*environmental perceptions*) and is lacking intrinsic motivation (*goal valuation*). The interplay of these different factors contributes to Thomas’ cognitive skills not being optimally used, and underperformance occurs.

## Conclusion and Discussion

The present study aimed at enhancing our understanding of inhibiting and facilitating factors for academic achievement of intellectually gifted students in the first and second grade of secondary education. The AOM has already proven its strengths in multiple studies (Rubenstein et al., 2012; Ritchotte et al., 2014; Siegle et al., 2014, 2017; Brigandi et al., 2018). By capturing the lived experiences of six intellectually gifted students in this study, we were able to get more insight into the complexity of the process of motivational development that leads to task engagement and (under)achievement as reported by respondents themselves. The insights gained through in-depth self-reported components and relationships provided further evidence of the mechanisms central in the AOM.

The first aim of this study was to identify the components central to the AOM in the students’ lived experiences. It is clear from this study that the different core themes positioned within the AOM were found to be present in the data collected from intellectually gifted students through in-depth self-reporting: self-regulation, goal valuation, environmental perception, and self-belief (see codes and sub codes in Table 2). The respondents elaborated on all components put forward in the AOM and tackling different subthemes within each theme. Only the aspect of self-efficacy or self-belief was addressed less frequently. We cannot make any clear statement about the underlying reason(s), but we can think about several possibilities. Self-belief, and more specific, self-efficacy is a theme that requires students to reflect upon their own cognitive talents and skills. Maybe the students do not like to brag about their cognitive talents or they may be too insecure to talk about this aspect of themselves. It is also possible that the respondents have never experienced a ‘challenging’ task or never have thought about ‘being smart’, therefore students are limited by their own self-insights. This probably also makes it difficult for them to answer. Another possibility is that self-efficacy becomes a more prominent theme only later on in their educational career, when students experience more school failures.

Second, we explored facilitating and hampering factors experienced by all students. As theoretically expected (Siegle and McCoach, 2005; Ritchotte et al., 2014; Siegle et al., 2017), we found in this study that self-control or self-monitoring (*self-regulation*) is an important factor of students’ academic attitude and success. There was a clear difference in the monitoring of delayed satisfaction. ‘Fun tasks’ were postponed by all high-performing students until less appealing tasks were completed, while all underachievers chose to complete the fun tasks first (direct satisfaction). Earlier research points to the importance of self-control behavior, and the risks of low self-control behavior (Mischel et al., 1989; Krueger et al., 1996; Zimmerman and Schunk, 2011). Researchers have found significant links between self-control and positive social and cognitive outcomes. Self-monitoring or self-control appears to be a good predictor for academic achievement (Pintrich, 2004; Zimmerman and Schunk, 2011; Vermunt and Donche, 2017). Additionally, for self-management skills *(self-regulation)*, the respondents’ answers aligned with the theory of the AOM: when a student has not been sufficiently challenged in the past, he cannot fall back on certain study strategies. Data revealed that the participating schools often offer training in self-regulated strategies. However, the respondents indicate that they do not want or need being taught these strategies in this way.

Previous research on the AOM already pointed out that a challenging environment is very important (Brigandi et al., 2018). In this study we also noticed that the way in which the respondents *perceive* their *environments* relates to their academic attitude and performance. Underachievers clearly perceived one or more environments (school, friends, home) as non-supportive. Well-performing students perceived most of their environments as supportive; only ‘school’ was sometimes mentioned as hampering. Both well- and underperforming students spoke of the hampering effect of the lack of interesting tasks at school. They define interesting tasks as tasks that address higher-order thinking skills and allow students to really learn, which can be linked to higher-level cognitive processes (Bloom et al., 1956). Interestingly, when talking about school, elementary education was mentioned several times. According to the students’ experiences, the transition to secondary education does not necessarily imply a more challenging, richer learning environment. Based on the interviews, elementary education is perceived as an environment that provides opportunities for creative, higher-order thinking. Secondary education appears to be more socially challenging (dealing with different teachers, attending a bigger school, making new friends,…). Even having an enrichment pullout program at secondary school is no guarantee of intellectual stimulation, according to the respondents in this study; the quality of the program is decisive.

It was striking that “achieving good grades” (*goal valuation*) was mentioned as a crucial motive for studying by the high-performing students. It is an interesting question why this aspect is so present. Is this encouraged in education? Is it because there is an absence of other motives such as intrinsically interesting or challenging tasks? Is it a reflection of students’ achievement motivation and performance orientation? Does this in the longer term lead to equally good talent development as other study motives (Pintrich, 2000; Ryan and Deci, 2000; Kyndt et al., 2015)? Further research is needed to answer these questions.

Another observation is that all respondents had both positive and negative intrinsic motivation experiences (*goal valuation*), regardless of their performance. The lack of motivation was, as expected (Whitmore, 1986; Snyder and Linnenbrink-Garcia, 2013), clearly present with the underperforming students. They also spoke, however, about their intrinsic motivation in the classroom. On the other hand, good performing students also frequently reported their lack of intrinsic motivation. Intrinsic value or motivation is acknowledged as an important factor in the development of students (e.g., self-determination theory, Ryan and Deci, 2000). In the case of these respondents, however, intrinsic value was not a determining impeding or facilitating factor in their motivation.

We illustrated how the interplay between the different aspects of the AOM is perceived by well achieving versus underachieving students. As stated in the AOM the well-performing students who show high levels of motivation generally have positive environmental perceptions, goal valuation and self-efficacy. Some components, however, seem to have a stronger influence on the students’ motivation than others. This is reflected in the discussed case of Nick; the well performing student, for whom all components were facilitating, only environmental perceptions had a minor impeding effect. A more complex reality is apparent when we look at the case of the underperforming respondent, Thomas. One aspect cannot be disconnected from another, as in this case the interplay of the lack of environmental support, intrinsic motivation and self-efficacy all influenced the motivation, task engagement and achievement of the respondent. By analyzing this case, we can point out the importance of taking into account the AOM in all its complexity, and not isolating one or more aspects.

When interpreting our findings, some limitations need to be taken into account. This study used the AOM as a theoretical lens. On the one hand, it may be too restrictive, because the four factors of the model (self-efficacy, goal valuation, environmental perceptions and self-regulation) were primarily considered, and may have discarded other possible motivational determinants. But on the other hand, the AOM is a broad and dynamic model that maps many different influencing factors. For future research it is interesting to further deepen the core concepts in the model (e.g., self-regulation).

Some methodological limitations are present in this study. First, we used a small purposeful sample. This, however, was a deliberate choice in order to get more in-depth insight from information- rich cases. The case-based approach of this study enabled to take into account the voice of the students, which is not always easy to capture. Longitudinal case study research using observation techniques may provide an even more in-depth picture of these reported realities of students. Second, five students were involved in the study because of their presumed intellectual giftedness, which had not been formally tested. This belief was grounded in conversations between the school counselor and the student, the parents and the teachers. Thus, multiple sources and actors were consulted before the students were identified as intellectual gifted. Nevertheless, it would interesting to also include a cognitive abilities test in further research in addition to nomination by counselors. Third, the socio-cultural background of the students was not assessed. We assume that students primarily came from middle class backgrounds, but assumption is based only on conversations with the students. It is recommended to assess the students’ socio-economic background in further research. Next, all respondents were male. Previous research has shown that there is a bias in the nomination of intellectually gifted students: boys are more likely to be nominated as cognitively gifted than girls (Bianco et al., 2011; Petersen, 2013; Lavrijsen and Verschueren, 2019).

Despite these limitations, this qualitative research is valuable for theory and practice. The components and the processes of the AOM appeared to be applicable in this specific educational context. Allowing students to speak for themselves opened up a source of information that should not be underestimated. Intellectually gifted students from the first and second year of secondary education have no problem to express their experiences, their frustrations and their needs very well. With this article, we want to emphasize the value of taking into account the perceived realities of respondents to obtain rich data both in scientific research and practice.

## Data Availability Statement

The datasets generated for this study are available on request to the corresponding author.

## Ethics Statement

This study was carried out in accordance with the recommendations of the guidelines for research ethics of the Declaration of Helsinki and the European General Data Protection Regulation (GDPR) and the Social and Societal Ethics Committee of the University of Leuven with written informed consent from all subjects. The protocol was approved by the Social and Societal Ethics Committee of the University of Leuven.

## Author Contributions

KB conducted the study. KV and VD contributed to the design of the study, discussed the interview coding, and participated in the writing of the manuscript.

## Conflict of Interest

The authors declare that the research was conducted in the absence of any commercial or financial relationships that could be construed as a potential conflict of interest.

## Footnotes

1 Educational tracks in secondary education in Flanders are based on students’ academic performance, with the academically oriented track having the highest academic performance standards. Accordingly, during secondary education more and more students change from the academic track to a less academically oriented track (i.e., technical or vocational track), a phenomenon called the “educational waterfall” (Dockx and De Fraine, 2019).
