# Supplementary material for: Academic (Under)achievement of Intellectually Gifted Students in the Transition Between Primary and Secondary Education: An Individual Learner Perspective
Source: Front Psychol. 2019 Nov 13;10:2533. doi: 10.3389/fpsyg.2019.02533 (PMC6863923; doi:10.3389/fpsyg.2019.02533)
Supplement: Supplementary file 1 [file Data_Sheet_1.docx]

**Appendix**

**Semi-structured interview** **schedule**

| [explaining procedure of the interview, signing informed consent] |
| --- |
| **Introduction**   - Can you introduce yourself? - What do you like about going to school? - What do you dislike about going to school? - Is school important for you? - Do you have a particular interest or passion? |
| **Self-beliefs**   - How would you describe yourself in class? - Are you satisfied with your work at school? Why (not)? - Which statement fits you best? Why?   - I prefer to work on a task that is more difficult.   - I enjoy working on a task at my level that I can complete without too much effort. - Do you think you can further develop your intelligence by going to school? Why?   - What do you think about being ‘smart’?   - Do you think you possess the necessary qualities to do good in class? |
| **Goal valuation**   - Do you find it important to do well at school? Why? - When do you find a task useful to make? - What is a condition for you to do engage in class? |
| **Self-regulation**   - Which statement fits you the most? Why?   - I prefer to first do a task that I enjoy and therefore I postpone the tasks that I do not like as much as possible.   - First I always make the least fun tasks and when they are done I can make the ones that I actually like. - Do you have a specific method for studying? If so, which one? - Can you sometimes work independently in the classroom? What are your experiences with this type of work? |
| **Environmental perceptions**   - Do you feel like your parents support you in studying? In which way? - Can you think of a situation when you were not at ease in the classroom? A threatening situation? How did you deal with this situation? (+ reversed question) - Do you find it important to have friends? Why? - Do you think motivation can be encouraged by a teacher? If so, how? |
| **General questions who are inclined to tackle one or more aspects of the Achievement Orientation Model**   - When you think about your courses in school. What motivates you? - What is your favourite subject? Why is this your favourite subject? - Can you think of a course that you originally didn’t like, but after a while you started to find the course interesting? Why did you became interested in it? + reversed question - Which card is the most important for you?   - SCHOOL-FRIENDS-HOBBY |
| **END**   - Can you think of a situation that causes you to feel sad in class? - Can you think of a situation that would make your heel happy in class? |
